# Purification, characterization and amino acid content of cholesterol oxidase produced by *Streptomyces aegyptia* NEAE 102

**DOI:** 10.1186/s12866-017-0988-4

**Published:** 2017-03-29

**Authors:** Noura El-Ahmady El-Naggar, Sahar F. Deraz, Hoda M. Soliman, Nehal M. El-Deeb, Nancy M. El-Shweihy

**Affiliations:** 1 Department of Bioprocess Development, Genetic Engineering and Biotechnology Research Institute, City of Scientific Research and Technological Applications, Alexandria, Egypt; 2 Department of Protein Research, Genetic Engineering and Biotechnology Research Institute, City of Scientific Research & Technological Applications, Alexandria, Egypt; 3 Department of Botany, Faculty of Science, Mansoura University, Mansoura, Egypt; 4 Biopharmacetical Product Research Department, Genetic Engineering and Biotechnology Research Institute, City of Scientific Research and Technological Applications, Alexandria, Egypt

**Keywords:** *Streptomyces aegyptia* NEAE 102, Cholesterol oxidase, Purification, DEAE Sepharose CL-6B, Characterization, Molecular weight, Amino acid contents

## Abstract

**Background:**

There is an increasing demand on cholesterol oxidase for its various industrial and clinical applications. The current research was focused on extracellular cholesterol oxidase production under submerged fermentation by a local isolate previously identified as *Streptomyces aegyptia* NEAE 102. The crude enzyme extract was purified by two purification steps, protein precipitation using ammonium sulfate followed by ion exchange chromatography using DEAE Sepharose CL-6B. The kinetic parameters of purified cholesterol oxidase from *Streptomyces aegyptia* NEAE 102 were studied.

**Results:**

The best conditions for maximum cholesterol oxidase activity were found to be 105 min of incubation time, an initial pH of 7 and temperature of 37 °C. The optimum substrate concentration was found to be 0.4 mM. The higher thermal stability behavior of cholesterol oxidase was at 50 °C. Around 63.86% of the initial activity was retained by the enzyme after 20 min of incubation at 50 °C. The apparent molecular weight of the purified enzyme as sized by sodium dodecyl sulphate-polyacryalamide gel electrophoresis was approximately 46 KDa. On DEAE Sepharose CL-6B column cholesterol oxidase was purified to homogeneity with final specific activity of 16.08 U/mg protein and 3.14-fold enhancement. The amino acid analysis of the purified enzyme produced by *Streptomyces aegyptia* NEAE 102 illustrated that, cholesterol oxidase is composed of 361 residues with glutamic acid as the most represented amino acid with concentration of 11.49 μg/mL.

**Conclusions:**

Taking into account the extracellular production, wide pH tolerance, thermal stability and shelf life, cholesterol oxidase produced by *Streptomyces aegyptia* NEAE 102 suggested that the enzyme could be industrially useful.

## Background

Cholesterol oxidase (EC 1.1.3.6) or 3ß-hydroxysteroid oxidase is a FAD (flavin adenine dinucleotide)-dependent enzyme belongs to the family of oxidoreductases, which is capable of catalyzing two reactions, oxidation and isomerization of cholesterol to give rise ketones (cholestenone) using molecular oxygen as an electron acceptor to form cholest-4-en-3-one and hydrogen peroxide [1].

Now cholesterol oxidase has considered as one of the most widely used enzymes in clinical laboratories as it participates in bile acid biosynthesis. The cholesterol oxidase enzyme exhibit many distinct properties; it is simple, specific and highly sensitive; its clinical applications has become widespread in the determination of serum cholesterol for the assessment of atherosclerosis, coronary heart disease and other lipid disorders and for determining the risk of heart attack and thrombosis. This enzyme shows biological insecticidal activity and it has been developed as a pest control in the agricultural industry especially in transgenic crops and as a potent parricide [2, 3]. Cholesterol oxidases can also be used to produce a precursor for chemical synthesis of steroid hormones [1] and to degrade of dietary cholesterol in foods [4].

The cholesterol oxidases are classified into two types (I and II) on the basis of the nature of the FAD linkages to the enzyme protein molecules. The type I and II involve non-covalent and covalent linkage of the FAD cofactors to the protein molecules, respectively [5]. The type I enzyme belongs to the glucose/methanol/choline (GMC) oxidoreductase family, while the type II enzyme belongs to the vanillyl alcohol oxidase (VAO) family [5]. Type I enzymes have been identified mostly in actinomycetes such as, *Streptomyces* sp. SA-COO, *Rhodococcus equi*, *Mycobacterium tuberculosis*, *Corynebacterium urealyticum* [6]. Although cholesterol oxidases of both classes share the same catalytic activity, they show significant differences in their redox and kinetic properties. In general, to exhibit a broad range of steroid specificities and capability of oxidizing a number of hydroxysterols, the presence of a 3β-hydroxyl group is an important requirement for their activity in all cases [7].

Cholesterol oxidases have been purified and characterized from several microorganisms belongs to diverse environments and their action is strongly influenced by the environment of its substrate [8]. According to Whitaker [9] the purified enzyme was characterized by determining the optimum pH and temperature for both activity and stability. The molecular weights of the cholesterol oxidases have been reported in the range of 36–61 kDa. Microbial cholesterol oxidases generally have neutral pH optima and possess stability over a wide range [10, 11].

The objective of this study is to purify and characterize the cholesterol oxidase produced in the fermentation medium of *Streptomyces aegyptia* NEAE 102.

## Methods

### Microorganisms and cultural conditions

*Streptomyces aegyptia* NEAE 102 [12] and other *Streptomyces* spp. used in this study are local isolates isolated from various soil samples which collected from different localities of Egypt and kindly provided by Dr. Noura El-Ahmady El-Naggar (Department of Bioprocess Development, Genetic Engineering and Biotechnology Research Institute, City of Scientific Research and Technological Applications, Alexandria, Egypt). These isolates were maintained on slopes containing starch-nitrate agar medium [13] of the following composition (g/L): Starch 20; KNO_3_ 2; K_2_HPO_4_ 1; MgSO_4_.7H_2_O 0.5; NaCl 0.5; CaCO_3_ 3; FeSO_4_.7H_2_O 0.01; agar 20 and distilled water up to 1 L. Slopes were incubated for a period of 7 days at 30 °C. The isolates were stored as spore suspensions in 20% (v/v) glycerol at −20 °C for subsequent investigation.

### Qualitative screening for cholesterol oxidase producing microorganisms using colony staining method

The principal of cholesterol oxidase activity is based on its ablitiy to convert cholesterol into cholest-4-en-3-one and hydrogen peroxide. Primary screening agar plate containing cholesterol as the sole carbon source was used. This experimental medium contained (g/L): Cholesterol 2, KNO_3_ 2, K_2_HPO_4_ 1, MgSO_4_.7H_2_O 0.5, NaCl 0.5, CaCO_3_ 3, FeSO_4_.7H_2_O 0.01, agar 20 and distilled water up to 1 L. Agar plates were seeded with spores of actinomycetes and incubated at 30 °C for a week. To confirm potentialities for cholesterol oxidase production, colony staining method was performed on the grown colonies. Filter paper discs were dipped into the assay solution containing 0.5% cholesterol; 6% phenol; 1.7% 4-aminoantipyrine and 3000U/l horseradish peroxidase in 100 mM potassium phosphate buffer pH 7.0 and were located on grown colonies on the plates and incubated for 24 h at room temperature. Penetration of cholesterol into the bacterial cell led to development of pink color in the medium surrounding the tested colonies due to the conversion of cholesterol into hydrogen peroxide and formation of quinoneimine dye as a result of cholesterol oxidase activity [14]. The strain which showed the most promising result was selected for further experiments.

### Inoculum preparation

The isolate was grown in 250 mL Erlenmeyer flasks containing 100 mL of broth medium consisted of (g/L: glucose 12; starch 9; yeast extract 6; peptone 4; (NH_4_) _2_SO_4_ 7.5; cholesterol 2; K_2_HPO_4_ 1; MgSO_4_.7H_2_O 0.5; FeSO_4_.7H_2_O 0.02; NaCl 1; MnSO_4_ 0.008; CaSO_4_ 0.002; ZnSO_4_ 0.002; CaCl_2_ 0.0002; Tween 80 0.05) [15]. Five disks of 9 mm diameter taken from the 7 days old stock culture grown on starch nitrate agar medium were used to inoculate the broth medium and grown at 30 °C with shaking (200 rpm) for 48 h and used as inoculum for subsequent experiments.

### Production of cholesterol oxidase by submerged fermentation

The previously prepared inoculums of the selected strain inoculated into 100 mL of fermentation medium dispensed in 250 mL Erlenmeyer conical flasks. The inoculated flasks were incubated on a rotatory incubator shaker at 150 rpm and 37 °C. After the specified incubation time for each set of experimental trials, the mycelium of each isolate was centrifuged with cooling (4 °C) at 5000 × g for 30 min. The cell free supernatant served as crude enzyme and was used for further investigations.

### Assessment of enzyme activity

Cholesterol oxidase activity of isolated bacteria was spectrophotometry determined by the modified method of Sasaki et al. [16] which based on generation of hydrogen peroxide during cholesterol oxidation reaction. This reaction was initialized by coupling hydrogen peroxide with 4-aminoantipyrine and phenol to produce quinoneimine dye followed by the measurement of absorption at 500 nm. The reaction mixture was composed of 3 μM of cholesterol in 1 mL of 1% Triton X-100 (used as substrate of the reaction), 0.1 mL of enzyme solution, 300 μM of potassium phosphate buffer pH 7.0, 1.2 μM 4-aminoantipyrine, 21 μM of phenol and 20 U of horseradish peroxidase in a final volume of 3 mL. The enzyme reaction was performed at 37 °C for 10 min with shaking along incubation period. To stop the reaction, the assay mixture was boiled for 3 min. One unit of enzymatic activity (U): was defined as the amount of enzyme required to form one micromole of H_2_O_2_ per minute at 37 °C.

### Purification of cholesterol oxidase from *Streptomyces aegyptia* NEAE 102

The purification was carried out using culture supernatant obtained after centrifugation at 11,000 × g for 30 min and used as crude enzyme extract. All purification procedures was performed at 4 °C. The supernatant was transferred into a conical flask, placed on the magnetic stirrer and subjected to ammonium sulphate precipitation by adding finely powdered ammonium sulphate pinch by pinch until complete dissolving and reaching 50% saturation and kept overnight in the refrigerator. The precipitate was then recovered by centrifugation at 11,000 × g for 30 min and the supernatant was further saturated up to 60–90% with ammonium sulphate. Then the precipitates were collected separately by centrifugation and dissolved in 100 mM potassium phosphate buffer (pH 7.0). The dissolved precipitated proteins were dialysed against the same buffer using a pre-treated dialysis tube (SERVA pro, 44144). Precipitate formed during dialysis was removed by centrifugation and was discarded. After dialysis, the samples were subjected to protein content estimation [17] and cholesterol oxidase activity determination. Active cholesterol oxidase fractions were pooled and stored at 4 °C for further purification. The concentrated enzyme solution was loaded on the column of DEAE-Sepharose CL-6B that was pre-equilibrated with 100 mM potassium phosphate buffer (pH 7.0) and the column was washed with the same buffer. The bound proteins were eluted with the same buffer containing 0.5 M of NaCl at a flow rate of 10 mL per 1 h. All chromatographic runs were monitored for protein estimation by reading the absorption at 280 nm. After fractions dialysis, the protein concentration was determined [17] and cholesterol oxidase activity was assayed. Fractions showing high cholesterol oxidase activity were collected for further use.

### Characterization of cholesterol oxidase enzyme

The effect of the incubation time on cholesterol oxidase activity was studied by incubating the reaction mixture for different times (15, 30, 45, 60, 75, 90, 105 and 120 min) and activity was quantified at different time intervals. To define the most functional pH of cholesterol oxidase activity; the purified enzyme was pre-incubated with different buffers over a range of pH 4–10 under assay conditions, and the residual activities of the enzyme was determined. The buffers, citric acid- Na_2_HPO_4_ (pH 4–6), 100 mM potassium phosphate (pH 7), Tris–HCl 0.05 M (pH 8) and glycine-NaOH (pH 9–10) were used. The influence of temperature on cholesterol oxidase activity was analysed by incubating the assay mixture over the temperature range of 25–60 °C in potassium phosphate buffer (100 mM, pH 7). Effect of different substrate concentration on cholesterol oxidase activity was measured  by incubating the enzyme with various concentrations of the specific substrate (0.05–5 mM) and then the enzyme activity was determined at each concentration.

### Determination of kinetic properties (K_m_, V_max_)

The reaction kinetics parameters of the purified enzyme was assayed by linear regression from Lineweaver-Burk plots [18, 19] with cholesterol as substrate under assay conditions. The Michaelis–Menten constant (K_m_) and maximal velocity (V_max_) were determined for the enzyme as function of temperatures using the Michaelis–Menten equation:

$$ V=\frac{V_{m ax}\ \left[ S\right]}{K_m + \left[ S\right]} $$

where V is the reaction velocity (a function of enzyme concentration), S is the concentration of the substrate, K_m_ is the substrate concentration at half-maximal reaction velocity, and V_max_ is the maximal velocity. V_max_ and K_m_ values were determined using nonlinear regression [19].

### Enzyme thermal stability

Thermostability of the cholesterol oxidase was carried by pre-incubating the buffered enzyme prepared in absence of its substrate for different time interval ranging from 0.0 to 90 min at various temperatures (50, 60, 70 and 80 °C). After incubation, the enzyme was cooled then the residual enzyme activities were determined under the defined conditions.

### Enzyme pH stability

The pH stability of the cholesterol oxidase was assayed after pre-incubating the enzyme in absence of substrate at 4 °C for 24 h at 3 h intervals in buffers of various pH values (pH 5, 7 and 10). The residual activity was assayed under the standard conditions.

### Polyacrylamide gel electrophoresis indicates a molecular mass of the purified cholesterol oxidase

To size and to determine the enzyme purity, SDS-PAGE (sodium dodecyl sulfate–polyacrylamide gel electrophoresis) was performed as described by Laemmli [20]. The gel system contained a separating acrylamide gel of 10% and stacking gel 5% with 0.1% SDS. After the electrophoresis, the gel was carefully removed from the glass plates and the proteins in the gel were stained in coomassie brilliant blue R-250 followed by distaining with a solution of methanol- acetic acid and water in the ratio of 4:1:5. The molecular mass of individual enzymes was determined by referring to wide range molecular weight protein marker (molecular mass range: 9–178 kDa).

### Determination of amino acids content

Amino acid content of the purified cholesterol oxidase was carried out with Sykam amino acid analyzer at the Central Laboratory, City of Scientific Research and Technological Applications, Alexandria, Egypt. Two mL of the purified enzyme was hydrolyzed in 6 N HCl at 110 °C for about 8–12 h. After removal of hydrochloric acid at 40–60 °C, the hydrolysate was dissolved in 1 mL water and dried three consecutive times to remove most of the HCl. The hydrolysate was dissolved in sample dilution buffer and was loaded into the cation separation column (LCA K06/Na, 4.6 × 150 mm; Sykam GmbH, Eresing, Germany) and analyzed with the Sykam amino acid analyzer. Free amino acids were determined using an ammonia filtration column (LCA, K04/Na, 4.6 × 100 mm, Sykam, GmbH, Eresing, Germany) equipped with an automatic amino acid analyzer (Sykam).

## Results and discussion

**Fig. 1:**
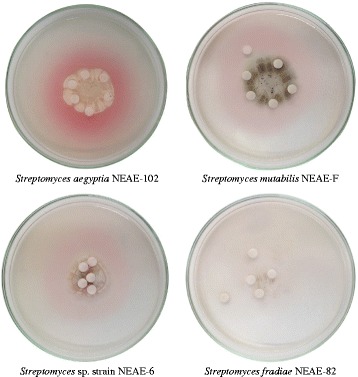
Cholesterol oxidase activity of different actinomycete strains detected by plate assay method. Development of pink color in the medium surrounding the colony due to the formation of quinoneimine dye indicated the presence of cholesterol oxidase activity; *Streptomyces fradiae* strain NEAE-82 cannot produce cholesterol oxidase activity

Different actinomycete strains were screened for their cholesterol oxidase activity using plate method, formation of pink zones around the colonies indicated the presence of cholesterol oxidase activity (Fig. 1). The promising isolate, *Streptomyces aegyptia* NEAE 102 was selected for further studies.

### Purification of cholesterol oxidase from *Streptomyces aegyptia* NEAE 102

**Fig. 2:**
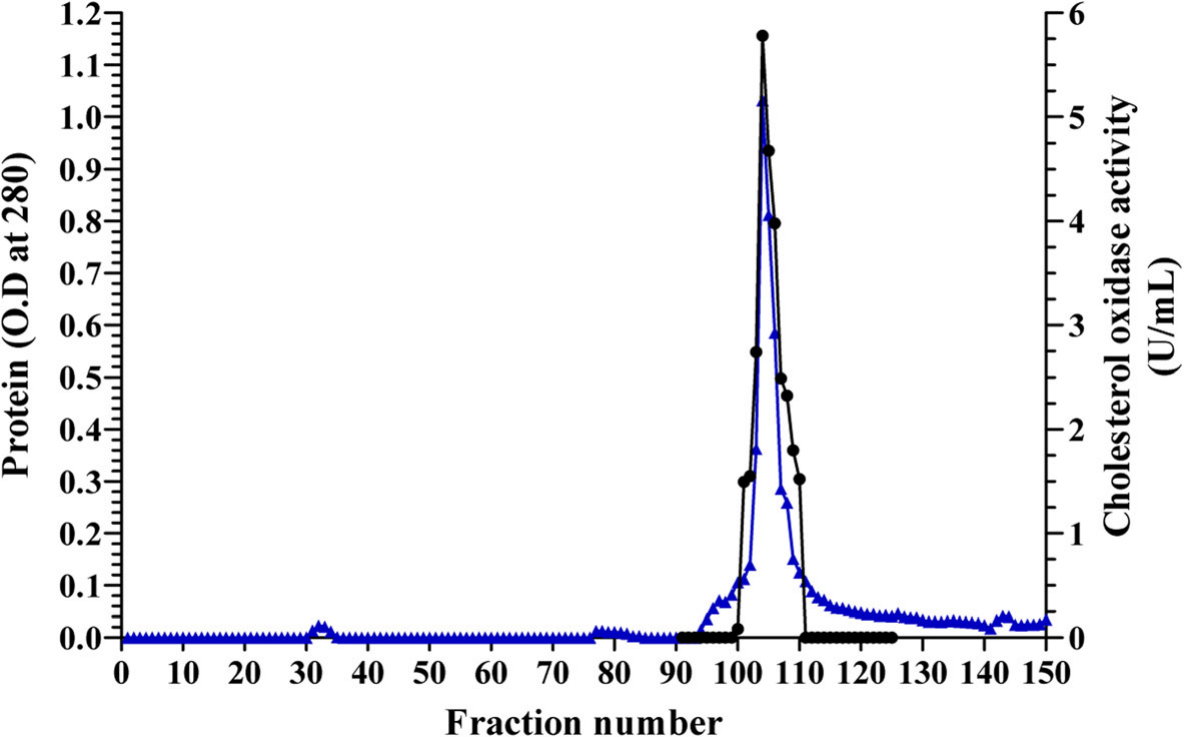
Purification of cholesterol oxidase produced by *Streptomyces aegyptia* NEAE 102 using ion exchange on DEAE Sepharose CL-6B. (▲) refer to protein, (●) refer to cholesterol oxidase activity

The crude culture filtrate of *Streptomyces aegyptia* NEAE 102 had a total activity of 19527.53 U with protein content 3811.27 mg; the specific activity was 5.12U/mg protein. The ammonium sulphate concentrated enzyme preparation had a protein content of 60.57 mg with specific activity of 20.11 U/mg protein, showing purification fold of 3.92. The enzyme recovery at this step was 6.23%. The fractions collected after ammonium sulphate precipitation were loaded on the column packed with DEAE Sepharose CL-6B and fractions each containing 2 mL were collected and analyzed for both enzyme activity and protein content for each separate fraction. The elution pattern obtained using DEAE Sepharose was graphically illustrated in Fig. 2. A total of 150 fractions were collected that showed one major cholesterol oxidase activity peak on the chromatogram. Cholesterol oxidase was purified to homogenity after ion exchange column separation contained DEAE Sepharose CL-6B with 501.05 U total activity and 31.15 mg protein. The enzyme was purified with specific activity of 16.08 U/mg of protein. Summary of the purification steps of the cholesterol oxidase produced by *Streptomyces aegyptia* NEAE 102 is presented in Table 1.

**Table 1:** Summary of the purification steps of the cholesterol oxidase produced by *Streptomyces aegyptia* NEAE 102

Purification step	Total protein content (mg)	Cholesterol oxidase activity			
		Total activity (U)	Specific activity (U/mg protein)	Recovery (%)	Purification fold
Culture filtrate	3811.27	19527.53	5.12	100	1
(NH_4_) _2_SO_4_, post dialysis	60.57	1217.88	20.11	6.23	3.92
Ion exchange on DEAE Sepharose CL-6B	31.15	501.05	16.08	2.56	3.14

### Physico-chemical characteristics of cholesterol oxidase

Cholesterol oxidases from several microorganisms from various species have been isolated, purified and characterized. The activity of cholesterol oxidase of *Streptomyces aegyptia* NEAE 102 was evaluated at various levels of temperature, pH, effect of substrate concentration and incubation time.

### Effect of incubation time on cholesterol oxidase activity

**Fig. 3:**
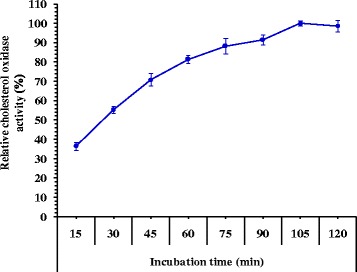
Effect of different incubation periods on cholesterol oxidase

The cholesterol oxidase activity (Fig. 3) increased as the incubation time increased up to 105 min (190.98 U/mL). Time beyond 105 min has slight decrease the activity of the enzyme (188.49 U/mL at 120 min of incubation).

### Effect of temperature on cholesterol oxidase activity

**Fig. 4:**
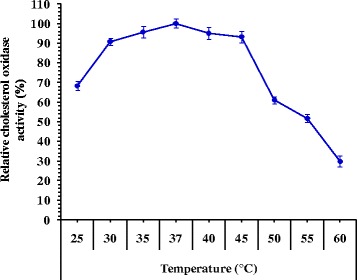
Effect of the temperature on cholesterol oxidase activity

The temperature optimum of cholesterol oxidase from *Streptomyces aegyptia* NEAE 102 is shown in Fig. 4. It showed marked activity with range of temperatures between 25 and 45 °C. The maximum cholesterol oxidase activity of 230.6 U/mL was obtained at 37 °C and at higher temperature the activity was declined. At temperature of 45 °C, the enzyme retained its 93.18% relative activity to the optimal activity. The enzyme retains 61.06% of its activity at 50 °C. The temperature ranges of notably cholesterol oxidase activity reported from different sources were 30–70 °C [10, 21, 22]. Most cholesterol oxidases exhibit optimum temperatures for enzyme activity in the range of 50–60 °C [23]. The optimum temperature of cholesterol oxidase activity from *Rhodococcus* sp. GKI was found to be around 30 °C. At temperature of 45 °C, the enzyme retained its 92% relative activity to the optimal activity [24]. Praveen et al. [22] reported that the optimum temperature of cholesterol oxidase from *Streptomyces parvus* at pH 7.2 was 50 °C. The cholesterol oxidase from *Streptomyces fradiae* is the most thermo-tolerant reported so far with optimum temperature of 70 °C [25]. Optimum temperature for cholesterol oxidase activity produced by *Pseudomonas aeruginosa* was determined by Jasim and Diwan [26] and the results showed that the optimum temperature for enzyme activity was 35 °C when the enzyme was added to cholesterol solution and incubated at different temperatures ranged between 25 and 65 °C for 10 min. At this temperature, the activity was 3.48 U/mL, and represents the optimum for enzyme activity because of the high effect on the reaction energy for both enzyme and substrate which leads to formation of enzyme–substrate complex and this will result in increasing the reaction speed as it was mentioned by Urban et al. [27], hence enzyme activity was decreased above and below this temperature. Most enzymes are often highly sensitive to high temperatures, while others which contain disulfide bonds are more stable in high temperatures than complex enzymes with high molecular weights as it was described by Segel [28].

Other studies reported that the temperature of 53, 47 and 40 °C were optimal for cholesterol oxidase activity from *Brevibacterium* sp. [29], *Rhodoccocus equi* and *Corynebacterium cholesterolicum* [30]; respectively. While Yazdi and Zahraei [25] reported optimum temperature at around 50 °C and plateau between 40 and 60 °C for the purified enzyme from *Streptomyces violascens*. Whereas, other authors reported that the optimum temperature for cholesterol oxidase from *Bacillus* sp., *Arthrobacter simplex* and *Streptomyces violascens* was found to range between 50 and 60 °C and obvious enzyme thermostability [31]. The optimum temperature for cholesterol oxidase was found to be 50 °C and it retained more than 70% activity at 50 °C after 60 min of heat treatment [32]. The optimal reaction temperature for cholesterol oxidase was 45 °C; it retained more than 80% activity in the temperature range 35–48 °C [33]. Comparing the effect of different temperatures on enzyme activity under a standard condition, cholesterol oxidase showed a marked activity between 20 and 50 °C with maximum activity at 35 °C [14]. Varma and Nene [34] found that cholesterol oxidase in the clarified fermentation broth have almost full activity at 45 °C for 1 h; at 50 °C with retained activity of 50%.

### Effect of pH on cholesterol oxidase activity

**Fig. 5:**
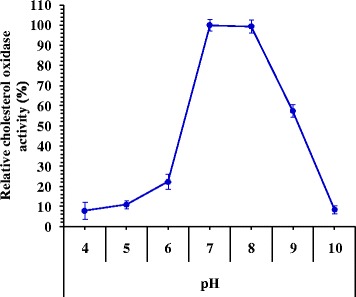
Activity of cholesterol oxidase as a function of the pH of the reaction

Cholesterol oxidase activity was studied as a function of pH in range between 4 and 10 (Fig. 5). The maximum enzyme activity at pH 7 is 237.31 U/mL (relative activity, 100%). At lower and higher pH’s, enzyme activity was decreased. The enzyme retains 99.44% of its activity at pH 8 (235.99 U/mL). However, the enzyme retains 57.59 and 10.98% of its activity at pH 9 and pH 5, respectively. Cholesterol oxidases from various microbial sources generally characterized to have neutral pH optima around pH 7.0 to 7.5 with stability over a wide pH range [10, 23]. The cholesterol oxidase from *Streptomyces parvus* exhibited maximum activity at pH 7.2 [22], while pH 7.5 was optimum for the enzyme from *Brevibacterium* sp. [29], that slightly decreased to 80% at pH range of 6.0–8.7. Jasim and Diwan [26] reported that the optimum pH for cholesterol oxidase activity was pH 7.0, when the purified enzyme was added to cholesterol solution incubated previously at a range of pH between 5.0 and 9.0 for 10 min at 32 °C. At this pH, enzyme activity was 3.8 U/mL, and the activity was decreased at the acidic and basic pH values because of the conformational changes in enzyme configuration due to the changes in the ionizable groups located in the active sites of the enzyme as it was mentioned by Whitaker [9]. Similar result was reported for the cholesterol oxidase from *Streptomyces* sp. which was most active at pH 7.0 [35]. The optimum pH of cholesterol oxidase reported from different sources were ranged from 5.0 to 8.5 [10, 21, 22]. The optimal pH for cholesterol oxidase was 7.5 and retained more than 80% activity in the pH range of 6.5–9.0 [33]. The best pH for cellular cholesterol oxidase enzyme produced by *Rhodococcus* sp. was pH 7.5 [14]. Cholesterol oxidase is pH dependent with maximum activity at pH around 7.0. Enzyme activity was found to be stable for at least 60 min at pH 7.0 by exhibiting more than 80% activity [32].

### Effect of substrate concentration on the activity of cholesterol oxidase

**Fig. 6:**
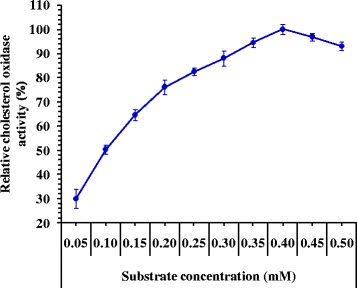
Effect of the substrate concentration of the reaction on cholesterol oxidase activity

The influence of substrate concentration on cholesterol oxidase activity was examined at different concentrations of substrate ranging from 0.05 to 0.5 mM to determine the optimum concentration required to give the highest cholesterol oxidase activity. The results in Fig. 6 showed a gradual increase in the enzyme activity with the increase in substrate concentration from 0.05 to 0.4 mM. However, further increase in substrate concentration (0.45 to 0.5 mM) lead to decrease in enzyme activity to 93.03% with 0.5 mM substrate. The optimum substrate concentration for maximum cholesterol oxidase activity was observed at 0.4 mM (423.3 U/mL).

**Fig. 7:**
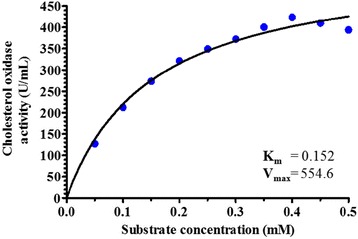
Michaelis-Menten plot for cholesterol oxidase produced by *Streptomyces aegyptia* NEAE 102

The typical Michaelis-Menten relationship was obtained between the substrate concentrations and the initial velocity of the reaction. Michaelis-Menten plot showed in Fig. 7 illustrated the K_m_ and V_max_ values for cholesterol oxidase enzyme. The plot gave K_m_ value of 0.152 mM and V_max_ of 554.6 U/mL for the hydrolysis of cholesterol. K_m_ value is defined as the concentrations of substrate that permits the enzyme to achieve half of the maximum reaction rate (V_max_) which implies that half of the enzyme active sites in the sample are filled (i.e. saturated) by substrate molecules in the steady state. K_m_ provides useful information regarding the affinity of the enzyme for its substrate [36]. The enzyme with a low K_m_ has strong binding affinity between the enzyme and substrate. V_max_ is expressed the maximum velocity of the reaction that reveals the turn over number of an enzyme which is the number of molecules of substrate converted into product by one enzyme site per second. If the molar concentration of enzyme is known, V_max_ be expressed as moles of product formed per second per mole of enzyme sites [37]. However, there are many factors that affect on kinetic parameters of the enzymes (K_m_ and V_max_) such as; type of enzyme, different forms of enzyme (crude, modified or purified), changes in enzyme conditions (pH, temperature, etc.), source of the enzyme (different microorganisms), type of used substrates and the assay procedures [38].

Doukyu et al. [39] determined the K_m_ and V_max_ values of cholesterol oxidases from various bacterial origins. The K_m_ values were 26.2, 18.4, 18.8, 76.8, 183 and 315 μM from *Chromobacterium* sp. DS-1, *Nocardia* sp., *N. erythropolis*, *B. cepacia*, *P. fluorescens* and *Streptomyces* sp., respectively. Thus, the K_m_ values of cholesterol oxidase from *Streptomyces aegyptia* NEAE 102 is relatively lower than those of *B. cepacia*, *P. fluorescens* and *Streptomyces* sp.

### Thermal stability

**Fig. 8:**
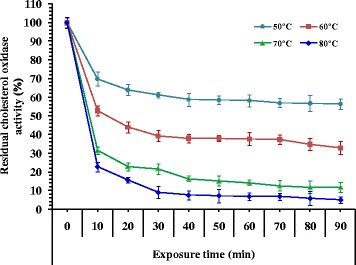
Thermal stability of cholesterol oxidase as a function of the time of the reaction

The heat stability of cholesterol oxidase is of a great advantage for clinical use [31]. The effect of temperature on the stability of cholesterol oxidase showed maximum enzyme activity at 50 °C (Fig. 8). Around 63.86% of the initial activity was retained by the enzyme after incubation at 50 °C for 20 min. Increasing exposure time up to 90 min resulted in about 43.64% of the enzyme activity, while a rapid decrease in the enzyme activity (6.68%) was observed after incubation at 80 °C for 60 min. Thermal inactivation process of the enzyme follows the theoretical curve of a sample first order reaction. However, linear regression of the obtained data was assayed to determine half-life time (T_1/2_) as shown in Table 2. The half-life time (T_1/2_) was 156.25 min at 50 °C, while being 107.27 min at 60 °C. On the other hand, destruction of enzyme activity was observed at 80 °C with low half-life time (80.33 min). It can be concluded from the previous results that the higher thermal stability behavior of cholesterol oxidase was at 50 °C. Results presented in Table 2 were normalized to activity of un-incubated enzyme (time 0 = 100%) for the percentage of activity remaining. Heat inactivation half-life (T_1/2_) and heat deactivation constant (*k*) were determined by fitting the data to a first-order decay curve using Graph-Pad Prism software.

**Table 2:** Half life time (T_1/2_) and heat deactivation constant (k) of cholesterol oxidase produced by *Streptomyces aegyptia* NEAE 102

Half life time (min)	
50 °C	156.25
60 °C	107.27
70 °C	81.82
80 °C	80.33
Thermal inactivation rate constant *k*_*d*_ *1* (min^−1^)^a^	
50 °C	0.0044
60 °C	0.0065
70 °C	0.0085
80 °C	0.0086
D-value (min)	
50 °C	281.25
60 °C	193.09
70 °C	147.28
80 °C	144.60
Thermal inactivation rate constant *k*_*d*_ *2* (min^−1^)^b^	
50 °C	0.0025
60 °C	0.0036
70 °C	0.0047
80 °C	0.0048

^a^ *k*_*d*_ *1* is the deactivation constant after losing 50% of initial activity (at T_1/2_)

^b^ *k*_*d*_ *2* is the deactivation constant after losing 90% of initial activity (at D- value)

Elalami et al. [24] reported that cholesterol oxidase from *Rhodococcus* sp. GKI was stable at the temperatures of 20, 30 and 40 °C for 2 h while, the half-life of cholesterol oxidase activity at 50 °C was around 70 min. Cholesterol oxidase obtained from *Rhodococcus* sp. R14-2 retained its maximum activity for 30 min at 50 °C but at temperatures higher than 60 °C, enzyme significantly inhibit its activity and at 70 °C was exhibited no activity within 1 h [21]. A highly thermo-stable cholesterol oxidase has been reported from *Chromobacterium* sp. strain DS-1 in comparison to the cholesterol oxidases obtained from various microbial sources of diverse environments such as *Nocardia* sp., *Pseudomonas fluorescens*, *Streptomyces* sp., *Nocardia erythropolis*, *Cellulomonas* sp. and *B. cepacia* ST-200 [39]. Similar result was reported for all of these commercial enzymes which suppress most of their activity after incubation at 60–80 °C for 30 min [39, 40]. Praveen et al. [22] reported that cholesterol oxidase from *Streptomyces parvus* did not lose its activity from 4 to 65 °C and exhibit optimum activity at temperature 50 °C with a pH value of 7.2. However, activity comparison after incubation for 30 min at 55, 60 and 65 °C showed retained activity about 68, 66 and 46%, respectively and lost almost all activity (86%) after 30 min at 75 °C.

Cholesterol oxidase of *Streptomyces virginiae* showed marked stability below 40 °C without notable inactivation after incubation of the enzyme for 6 h. However, cholesterol oxidase activity rapidly declined at temperature exceeded 50 °C with retained activity of approximately 40 and 17% of its initial activity after incubation for 6 h at 50 °C and 1 h at 60 °C, respectively [33]. The commercially available cholesterol oxidases from different microbial sources such as *Streptomyces* sp., *Pseudomonas fluorescens, Cellulomonas* sp., *B. cepacia* ST-200, *Nocardia erythropolis* and *Nocardia* sp.[41] exhibit complete lose of their activity after incubation at 60–80 °C for 30 min [39, 40]. But, the enzyme from *Streptomyces aegyptia* NEAE 102 retained 39.16% of its original activity at 60 °C after 30 min.

A multiple mutant (R135H, S103T andV121A) showed the highest half life stability of 52.2 min at 60 °C compared to wild type which had half life stability of 7.8 min [41]. The optimum stability of cholesterol oxidase produced from *Streptomyces fradiae* is reported at 70 °C with complete retained activity at 50 °C for 90 min [25]. Moreover, almost full enzyme activity was observed at temperatures up to 40 °C and pH 7.5 for 1 h. However, the enzyme retained up to 65 and 50% of its activity at temperatures of 45–50 °C for 30 min and 1 h, and only 20% of the enzyme was active for 30 min at 55 °C [34]. Cholesterol oxidase of *Pseudomonas* sp. showed stability at temperatures from 4 to 50 °C. However about 73% of its activity was retained after the incubation at 60 °C, and almost complete loss of activity at 70 °C [42].

### The storage life of cholesterol oxidase enzyme

The effect of different storage periods on the stability of cholesterol oxidase are shown in Table 3. Around 71.65% of the initial activity was retained by the enzyme after 7 days of storage at 4 °C, while a rapid decrease in the enzyme activity (17.41%) was observed after storage at room temperature for the same period of time (7 days). Cholesterol oxidase enzyme from *S. lavendulae* NCIM 2421 kept in a sterile container at room temperature had a storage life of 5 days and full retained enzyme activity when stored at 4 °C for 60 days with half-life of 105 days [34]. However, cholesterol oxidase enzyme from *S. parvus* had storage life of 6 months at 4 °C [22].

**Table 3:** The storage life of cholesterol oxidase produced by *Streptomyces aegyptia* NEAE 102

Storage period (Day)	Residual cholesterol oxidase activity (%)	
	At 4 °C	At room temperature
0	100	100
1	95.19	49.78
2	85.06	32.48
3	80.00	27.90
4	78.99	23.66
5	77.97	18.30
6	74.18	17.86
7	71.65	17.41

### pH stability

**Fig. 9:**
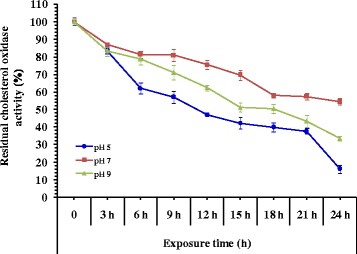
pH stability of cholesterol oxidase as a function of the time of the reaction

The effect of pH on the stability of cholesterol oxidase exhibited optimum enzyme activity at pH 7 (Fig. 9). At this pH, the enzyme activity was more stable, the remaining activity was 86.96 and 54.48% after 3 h and 24 h. Slight decrease was observed by increasing pH up to 9 for 3 h (83.31%). At pH 5, the enzyme retained 82.36% of its activity after 3 h and losing most of its activity (15.95%) after 24 h.

The cholesterol oxidase from *Streptomyces* sp. had an optimum pH for enzyme activity at pH 7.0 and 80% of its activity was retained at pH 6–8 for 24 h, but activity rapidly declined at higher or lower pH, and at pH 10, only 10.16% of its activity was retained [35]. On the other hand, it was found that the optimum pH for the stability of cholesterol oxidase produced by *Pseudomonas aeruginosa* was pH 6.5. At this pH, the enzyme activity was 3.62 U/mL, and the remaining activity was 100% [26]. The cholesterol oxidase from *Streptomyces parvus* had optimum activity at pH 7.2 and exhibited activity stability within a broad pH range of 4.0–11. However 40% of its activity was lost at pH 11 for 30 min [22]. Comparable pH stability was also reported by Varma and Nene [34] in which cholesterol oxidase showed stable activity values in the pH range of 6–10 for 1 h at 30 °C, but it lost 60% of its activity below pH 4 and above pH 10. A further declination of activity (11–17%) was reported in the range of pH 5–8 after incubation at 4 °C for 24 h and 60% of the activity was retained above this range. At pH 4 the enzyme was almost completely inhibited. Cholesterol oxidase from *Streptomyces fradiae* [25] was stable in the pH range 4–10 at 4 °C for 4 h.

### Molecular weight determination by Sodium Dodecyl Sulphate- Polyacrylamide Gel Electrophoresis

**Fig. 10:**
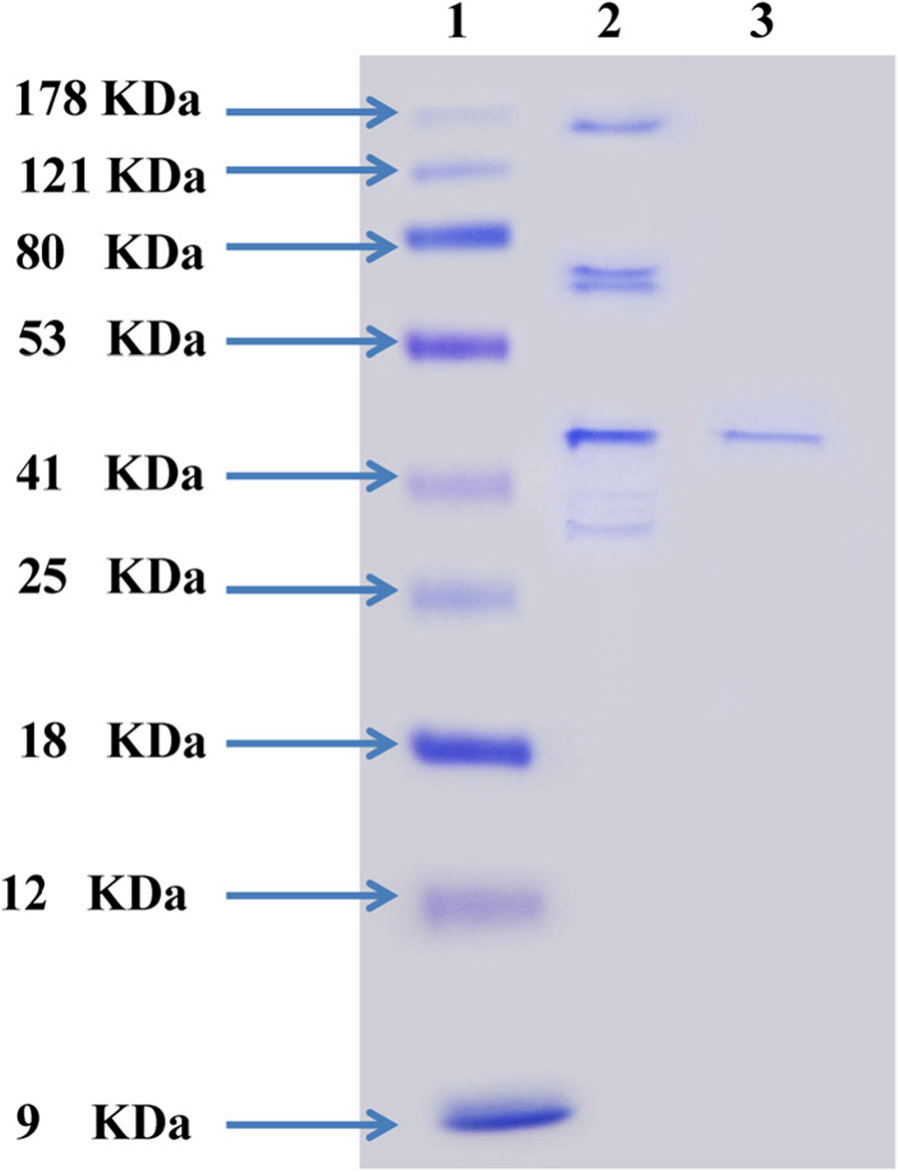
SDS-polyacrylamide gel electrophoresis of the purified cholesterol oxidase protein from *Streptomyces aegyptia* NEAE 102. The gel was stained with coomassie brilliant *blue* R-250. Lane 1: Protein marker; Lane 2: Ammonium sulphate fractions (60%); Lane 3: Purified cholesterol oxidase protein

The molecular weight of the purified cholesterol oxidase released from the final purification step on DEAE Sepharose was determined by SDS-PAGE according to the method of Laemmli [20]. The electrophoretic mobilities versus logarithmus of molecular weights of standard proteins (molecular mass range: 9–178 kDa) were photographed in Fig. 10. SDS–PAGE of the enzyme preparation revealed only a single distinctive protein band for the pure preparation of cholesterol oxidase with an apparent molecular weight of 46 kDa (Fig. 10). The molecular weights of the cholesterol oxidases have been reported in the range of 47–61 kDa [10]. Two novel extracellular cholesterol oxidases designated CO1 and CO2, from *Bacillus* sp. SFF34, were purified giving molecular weights values of 36 and 37 kDa [11]. The molecular weight of the obtained cholesterol oxidase was concluded to be 20.1 kDa [32].

### Amino acid composition

**Fig. 11:**
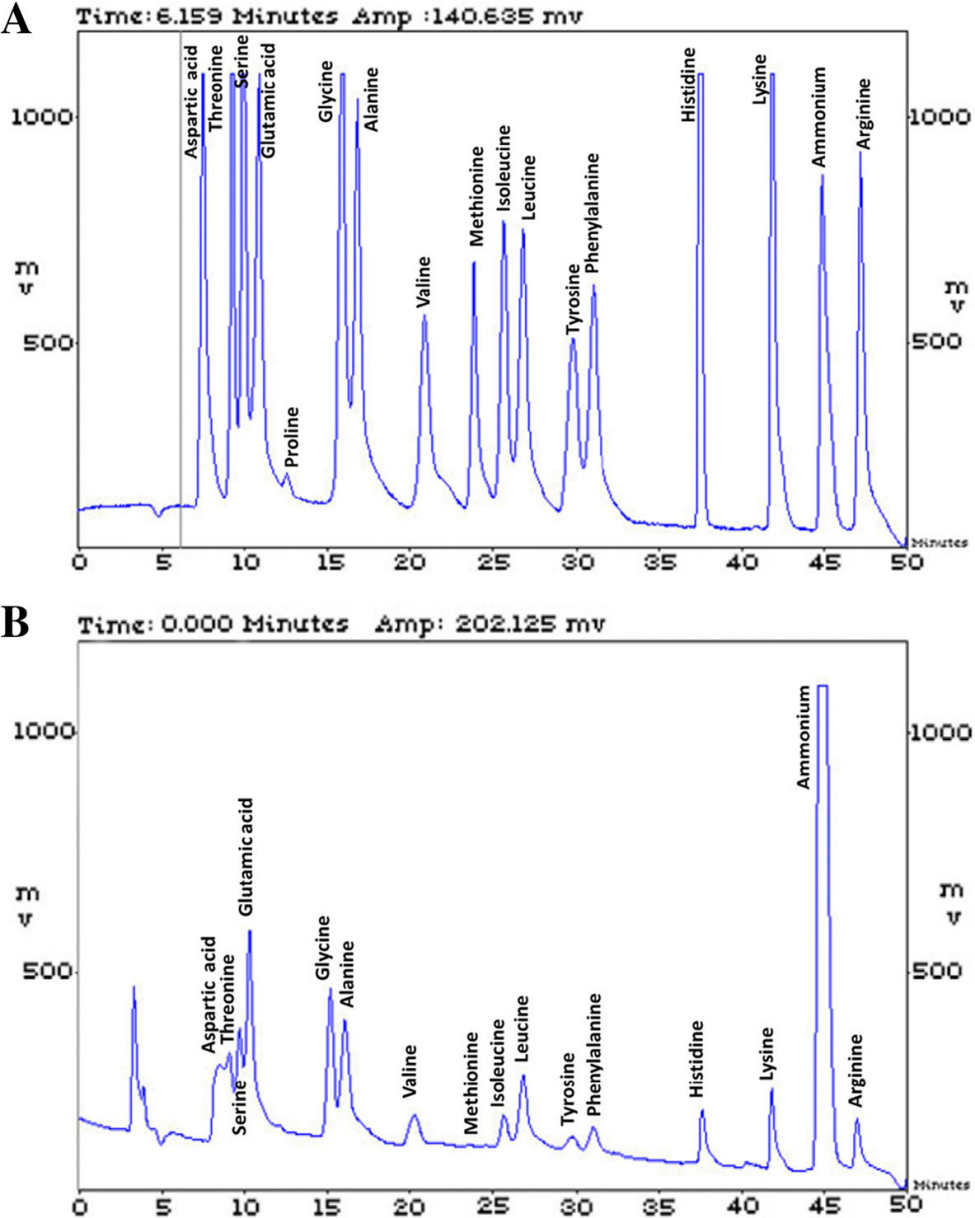
**a** Chromatogram of the amino acid standard mixture; **b** the chromatogram of *Streptomyces aegyptia* NEAE 102 cholesterol oxidase

Table 4 and Fig. 11 shows the amino acid contents of the purified cholesterol oxidase produced by *Streptomyces aegyptia* NEAE 102. Glutamic acid was the most represented amino acid in the quantified cholesterol oxidase (11.49 μg/mL). Relatively higher amounts of leucine, arginine, alanine were present. There are 20 commonly occurring amino acids in proteins which are found either in the free state or as linear chains in peptides and proteins. Amino acid analysis has an important role in the study of the composition of proteins, foods and feedstuffs. Free amino acids are also determined in biological material, such as plasma and urine, and in fruit juice and wine. When it is performed on a pure protein, amino acid analysis is capable of identifying the protein and the analysis is also used as a prerequisite for Edman degradation and mass spectrometry and to determine the most suitable enzymatic or chemical digestion method for further study of the protein. It is also a useful method for quantitating the amount of protein in a sample and can give more accurate results than colorimetric methods [43].

**Table 4:** Amino acids content of purified cholesterol oxidase produced by *Streptomyces aegyptia* NEAE 102

Amino acid	Residue concentration				Estimated no. of residues/mol^a^ (to nearest integer)
	μg/ml	nmol/ml	g/mol	Mol.%	
Hydrophobic					
*Aliphatic*					
Glycine (Gly)	5.53	73.71	75.06	10.76	39
Alanine (Ala)	7.77	87.26	89.09	12.73	46
Isoleucine (Ile)	3.46	26.40	131.17	3.85	14
Leucine (Leu)	10.28	78.71	130.67	11.49	42
Valine (Val)	3.66	31.21	117.14	4.55	16
*Aromatic*					
Phenylalanine (Phe)	5.71	34.54	165.17	5.04	18
Charged					
*Acidic*					
Aspartic acid (Asp + Asn)	5.62	42.24	133.00	6.16	22
Glutamic acid (Glu + Gln)	11.49	78.12	147.11	11.40	41
*Basic*					
Histidine (His)	5.91	38.10	155.17	5.56	20
Lysine (Lys)	5.48	37.46	146.18	5.47	20
Arginine (Arg)	8.67	49.79	174.19	7.27	26
*Polar*					
Serine (Ser)	4.31	40.96	105.10	5.98	22
Threonine (Thr)	5.27	44.26	119.11	6.46	23
Methionine (Met)	0.33	2.21	148.42	0.32	1
*Aromatic*					
Tyrosine (Tyr)	3.67	20.28	181.16	2.96	11
Total					361

^a^The number of calculated amino acid based on: if the MW of the purified enzyme is 46000 Da and average MW of the 20 most common aa is 110 Da, the no. of aa will be approximately 46000/110 = 418aa. Trp-residues cannot be determined, based on the methodology used. Pro residues were also not detectable. Because of the deletion of 2 of the 20 amino acids in the calculation: W, P (=10%), we have taken away 10% of aa

## Conclusions

Different actinomycete strains were screened for their cholesterol oxidase activity using plate method. The promising isolate, *Streptomyces aegyptia* NEAE 102 was selected for further studies. The crude enzyme extract was purified using ammonium sulfate precipitation, dialysis and ion exchange chromatography using DEAE Sepharose CL-6B. The enzyme was purified 3.14-fold and showed a final specific activity of 16.085 U/mg protein. The molecular weight of the purified cholesterol oxidase was determined as 46 KDa. Glutamic acid was the most represented amino acid in the quantified cholesterol oxidase (11.492 μg/mL).

## Abbreviations

FAD: Flavin adenine dinucleotide
*k*: Heat deactivation constant
KDa: Kilodalton
K_m_: Michaelis–Menten constant
SDS–PAGE: Sodium dodecyl sulfate–polyacrylamide gel electrophoresis
T_1/2_: Half-life time
V_max_: Maximal velocity
